# 

**DOI:** 10.1192/bjb.2025.10200

**Published:** 2026-08

**Authors:** Fraser Walker, Graham Walker

**Affiliations:** 1 NHS Greater Glasgow and Clyde, NHS Scotland, Glasgow, UK; 2 School of Health & Wellbeing, University of Glasgow, Glasgow, UK

*Steve*, directed by Tim Mielants and starring Cillian Murphy as the title character, is a powerful exploration of institutional care, adolescent vulnerability and caregiver burnout. The film follows Steve, headteacher of a 1990s secure residential school for boys with complex needs, and unfolds over a single 24 h period. The film depicts the interplay between adolescent distress and staff exhaustion, giving a fascinating insight into the effects of caregiving under sustained strain. We write as psychiatry trainees working in the National Health Service in Scotland and note the relevance of the film to those working in psychiatry for several reasons. The film depicts multiple principal psychiatric themes, including adolescent depression, adult addiction and the emotional volatility of working in an under-resourced service. The film is adapted from Max Porter’s short novel *Shy*, named after one of the adolescents at its centre, and offers a brief yet penetrating glimpse into the hopelessness that engulfs him. We learn of Shy’s strained relationship with his mother and stepfather, who in one scene decided to cut all contact with him. The narrative follows a visiting film crew documenting life at the school, at one point asking staff and pupils in interviews to describe themselves in three words. Shy’s self-description is ‘depressed, angry and bored’, offering an insight into the anhedonia, irritability and emotional exhaustion he appears to experience within the care system.

Headteacher Steve’s own three words, ‘very, very tired’, speak to the moral injury and psychological fatigue of working within a chronically under-resourced system. Within the school we see staff trapped in permanent firefighting mode, breaking up fights and dealing with emotionally taxing situations. We hear of multiple budget cuts, culminating in a particularly poignant scene where the staff are informed that the school will be imminently closing down. One could draw a comparison with working in healthcare, where we face similar challenges, including resource cuts, waiting times and closure of facilities, and have an awareness of how these may adversely impact our patients.

In one scene a weary teacher describes herself as ‘part prison guard, part nurse, part battleaxe and part mummy’. This prompts reflection on the multidimensional challenges faced by those working in in-patient settings and secure care facilities, where staff must take on multiple roles throughout a working day. Such dynamics are particularly evident if looked-after individuals lack a consistent social support network, and the film draws upon the importance of seeking help as professionals when we feel emotionally burnt out. As the story unfolds, Steve’s addiction gradually surfaces as a maladaptive response to manage this sense of overwhelming responsibility. We see a brief, surprising glimpse of his serene family life, highlighting the compartmentalisation professionals often adopt as a defence mechanism against the demands of work.

The film is gritty and raw, with handheld cameras capturing the chaos of life within the school. The soundtrack seems deliberately abrasive, with jittering drum and bass sounds set within the turmoil. As psychiatry trainees, we found *Steve* a particularly harrowing drama that drew on several of our experiences of and reflections from clinical practice. It is a reminder of the functional and psychological cost of under-resourcing both to staff and service users, and the fragility of working within the health and social care system.

